# Experimental Study on Flammability and Flame Spread Characteristics of Polyvinyl Chloride (PVC) Cable

**DOI:** 10.3390/polym12122789

**Published:** 2020-11-25

**Authors:** Weiguang An, Yanhua Tang, Kai Liang, Tao Wang, Yang Zhou, Zhijie Wen

**Affiliations:** 1 Jiangsu Key Laboratory of Fire Safety in Urban Underground Space, China University of Mining and Technology, Xuzhou 221116, China; ts18120098p31@cumt.edu.cn (Y.T.); TS17120070P3@cumt.edu.cn (K.L.); TS19120031A31@cumt.edu.cn (T.W.); 2 State Key Laboratory of Coal Resources and Safe Mining, China University of Mining and Technology, No. 1 University Road, Xuzhou 221116, China; 3 Key Laboratory of Mining Disaster Prevention and Control, Qingdao 266590, China; 4 School of Civil Engineering, Central South University, Changsha 410075, China; zyzhou@csu.edu.cn

**Keywords:** flammability, polyvinyl chloride, flame spread, flame size, temperature distribution, cable structure

## Abstract

Polyvinyl chloride (PVC) is widely applied in cables as insulation materials, which are vital for operation and control of industrial processes. However, PVC cables fires frequently occur, arousing public concern. Therefore, experimental methods are used to study flammability and flame-spread characteristics of PVC cable in this paper. Influences of cable structure and number are investigated, which is scanty in previous works. As cable core number of single cable or cable number of multiple cables rises, average flame height and width increase while the increment decreases. Formulas concerning dimensionless flame height and single cable diameter (or total width of multiple cables) are obtained. The former is negatively correlated with the latter. For single cable, convective heat transfer is dominant, and flame-spread rate decreases as cable core number increases. Cable maximum temperature, which drops first and then rises as cable core number increases, is observed in the cable core area. For multiple cable, the flame-spread rate increases as cable number increases. As the cable number rises, the length of pyrolysis and combustion zone increases while the maximum temperature of cable surface decreases. This work is beneficial to fire hazard evaluation and safety design of PVC cables.

## 1. Introduction

Cables are widely applied in people’s life and production. They are vital for operation and control of industrial processes [1]. The cable is comprised of four parts from the outside to the inside, i.e., the outer sheath, filler, insulating layer, and conductive core. Except the core material, above materials of the cable are carbon-based materials. Polyvinyl chloride (PVC) is widely applied in cables as the insulating layer, which are very susceptible to fire. This induces potential fire hazard because aging cable material, short circuit, and high-temperature environment will possibly cause the PVC insulation layer to be ignited. In ordinary life, electric fire accounts for approximately 60% of the total fire accidents [2]. In the chemical industry, cable fires may cause the explosion of chemicals. For thermal power plant, cable fires may lead to the fire and explosion of oil-immersed transformer. In nuclear power plant, cable fires may trigger the hydrogen explosion [3]. These accidents will lead to a lot of casualties [4]. In addition, once the cable fire occurs, cables and equipment will be destroyed, causing large-scale interruption of life or production, resulting in severe economic losses. Therefore, it is necessary to investigate the flammability and flame-spread characteristics of PVC cable.

Some works have been conducted concerning pyrolysis behavior, combustion property, and fire toxicity of cable materials. Experimental and numerical studies were conducted by Moinuddin et al. [5] on coupled solid-phase reactions (pyrolysis) and gas-phase reaction (combustion) for both charring and non-charring materials. Fernandez-Pello et al. [6] evaluated the fire resistance of cables commonly used in electrical facilities after obtaining the ignition time and fire-spread rate of the cables impacted by external radiant heat flux. Gong et al. [7] found that the ignition time of the cable grows linearly with the increase of the integral heat flux after uniformly heating a cable composed of PVC shell and an XPLE insulation layer in a new cylindrical heating chamber. Xie et al. [8] performed TG (thermogravimetric), FTIR (Fourier transform infrared spectrometry), and MCC (microscale combustion calorimeter) experiments in air and nitrogen environments respectively, and compared the fire performance of new and aged cables. They found that the pyrolysis and combustion of the aged cables were more complete. However, Wang et al. [9,10] investigated the pyrolysis and flammability of new and aged polyvinyl chloride (PVC) sheaths of cables. They found that an aged sheath generally pyrolyzes and combusts more weakly and incompletely compared to the new one. Beji et al. [11] performed a cable tray fire experiment to predict the heat release rate (HRR) curve based on the video fire analysis (VFA), and the obtained curve was consistent with the measured results. Matthias Siemon et al. [12] conducted large-scale cable tray fire experiments. They found that the longitudinal spacing of the cable tray had no significant impact on the mass loss rate of the cable. The tight arrangement of cables delayed the flame spread from bottom to top. Huang and Nakamura [13] conducted a review of fundamental combustion phenomena in wire and cable fires. The review emphasized the complex role of the metallic core in the ignition, flame spread, burning, and extinction of cable fire. Palin et al. [14] investigated effects of the poss-based nanoadditives on the thermal stability of cable-grade PVC. Moreover, hardness and mechanical properties were studied in order to highlight the effects of these additives in the perspective of final industrial uses. In Klapiszewski et al.’s work [15], preparation and characterization of eco-friendly Mg(OH)(2)/Lignin hybrid material were conducted and its use as a functional filler for PVC fire resistance was evaluated.

To reduce fire hazard of cables, some flame retardant [16,17,18,19,20,21] were used for thermosetting resins such as PVC. Nguyen et al. [16] studied the influences of clay and manufacturing on fire resistance of organoclay/thermoset nanocomposites and found the combination of mechanical and ultrasonic dispersing procedure had considerable influence on the nanoclay distribution. In addition, they developed a novel numerical procedure combining pyrolysis analysis of the organoclay-composites and the fire dynamic simulation of the combustion process to validate the thermal responses obtained from the cone calorimetry experiments [17]. Ferdous et al. [18] investigated the effect of ceram powder on the properties of composite laminates based on glass fibers and phenolic resin and found that while the increase of ceram decreased the strength properties of the composite laminates, the bulk density and bending modulus increased. Shan et al. [19] developed a new P-N containing the flame retardant, named N,N′-dibutyl-phosphate diamide (DBPDA), which could reduce the peak values of heat release rate and smoke production rate. Zhang et al. [20] prepared thermosetting composites based on cyanate ester (CE) and unique hybridized graphene oxide (FGO) with phosphorus and silicone, which had good performance concerning flame retardant, toughness, and heat-resistance. Song et al. [21] developed an integrated three-source intumescent flame retardant (IFR), which could decrease the peak of heat release rate by 43.45% and reduce the total heat release by 28.55%.

PVC cable structures and cable number are different for different application scenarios. Single cable structures and the number of multiple cables will significantly affect the flammability and flame-spread characteristics of PVC cables, resulting in the difference in fire hazards. However, from above literature review, it is found that there are fewer works concerning influence of cable structure and cable number. Therefore, it is necessary to conduct this work. Results obtained in this work are beneficial to fire hazard evaluation and safety design of PVC cables with different structures and quantity. The fire hazard evaluation will help in safety management and in conducting fire insurance of sites using PVC cables. Moreover, results of this work are helpful to determine the optimal single cable structure and the number of multiple cables to achieve the lowest fire hazard. In addition, this work provides reference to fire protection code of utility tunnels or other sites using PVC cables.

## 2. Experimental Materials, System, and Method

The experimental materials are PVC cables commonly used in the utility tunnel. The cable is comprised of four parts from the outside to the inside, i.e., the outer sheath, filler, insulating layer, and copper core. The material of the outer sheath is neoprene, and the inner filler is hemp rope (made of recyclable rubber). The material of the insulating layer is mainly polyvinyl chloride (PVC). The diameters of two-core, three-core, and four-core cables are 8.5 mm, 11 mm, and 12 mm, respectively. For cables with different cores, although the amount of cable fillers is different, the diameter of the copper core, the thicknesses of the outer sheath, and insulating layer are the same, which are 1.5 mm, 2 mm, and 1 mm, respectively. The physical parameters of the cable materials are shown in Table 1.

**Figure 1:**
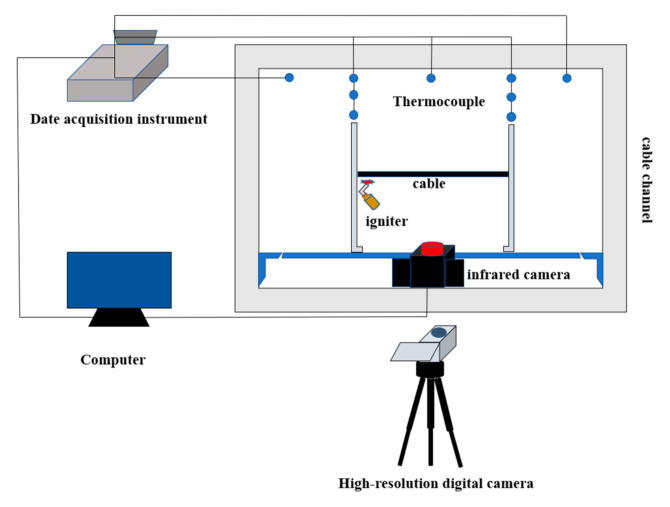
Schematic diagram of experimental system.

Experimental system and method in this work are relevant to ASTM E84 Tunnel Tests, which is a fire test standard for testing the combustion and flame-spread characteristics of building materials. In the ASTM E84 Tunnel Tests, fire spread rate and smoke concentration of building materials are measured, and the tested materials are divided into different grades according to their comprehensive performance. Similar experimental method is also used in Plumecocq et al.’s work [22]. The schematic diagram of the experimental system is presented in Figure 1. The system is composed of a cable channel, an experimental stent, experimental materials, and measuring instruments. The channel with a circular cross section is applied in this work. The length of the channel is 10 m, and the diameter of the cross section is 1.5 m. Natural ventilation is used in the experiments. The environmental conditions are as follows: atmospheric pressure is 100.07 kPa, ambient temperature is 30 °C, and humidity is 65%. The core number of a single cable and the number of three-core cables are changed in the experiments. The experimental conditions are shown in Table 2.

The measuring instruments applied in the experiment are high-resolution digital camera and infrared camera. The digital camera records the flame-spread process, which is stored in the computer. Processing with the computer program, the height and width of the flame could be obtained. The infrared camera is used to record the temperature change of the cable surface and its flame. It can take continuous shooting with a shooting frequency of up to 50 HZ. The infrared video is processed with a professional software, and then the change of pyrolysis front position of the cable over time is obtained to calculate the flame-spread rate. The ignition device is a card torch igniter fueled by butane with a volume concentration greater than 95%. The igniter is placed on the leftmost point of the cable. For multiple cables, multiple igniters were employed simultaneously to ensure that cables were ignited at the same time in the experiment. The combustion test under each experimental condition is repeated three times. The experimental errors of the average flame height of single, two, and three cables are 2.4%, 2.2%, and 3.7%, respectively, while experimental errors of the average flame width are 3.5%, 3.8%, and 4.3%, respectively. The experimental errors of the average flame height of two-core, three-core, and four-core cables are 2.1%, 2.4%, 6.8%, respectively, while the maximum errors of the average flame width are 5.6%, 3.5%, 4.7%, respectively. The experimental errors of the flame-spread rate of single, two, and three cables are 5.4%, 4.8%, 3.7%, respectively, while those of two-core, three-core, and four-core cables are 3.3%, 5.4%, 6.5%, respectively.

## 3. Results and Discussion

Experimental results of this paper mainly include the flame shape, flame size, flame-spread rate, and temperature distribution of the PVC cable. The flame size mainly includes flame height and flame width. Larger flame size leads to stronger heat radiation, and the combustible materials near the fire source will be ignited in a shorter time. Higher flame-spread rate means that the fire will spread to a wider area in a shorter time, increasing damaged area and the difficulty of rescue. Higher temperature corresponds to more heat released and longer cable preheating zone, which could increase the rate of flame spreading. In addition, more electrical equipment could be damaged owing to higher temperature. Therefore, larger flame size, higher flame-spread rate, and higher temperature correspond to higher fire hazard. Additionally, the effects of core number of a single cable and number of multiple cables on the experimental results are discussed.

### 3.1. Flame Shape and Flame Size

**Figure 2:**
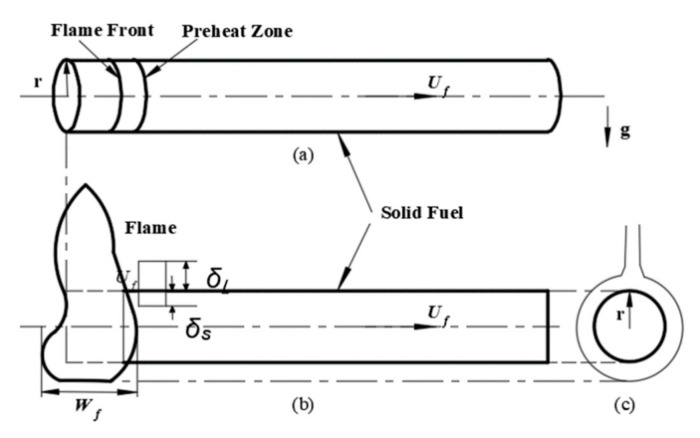
Schematic diagram of cable flame (**a**) flame front, (**b**) front view of the flame, (**c**) side view of the flame.

Figure 2 is a schematic diagram of the front view and side view of PVC cable flame. The flame wrapped the cylindrical material of the cable from the bottom surface to the top surface. The front area of the flame continuously conducts heat transfer in both solid phase and gas phase. Compared with the flat materials, the heat transfer coefficient is higher. When the combustion surface generates a heat flow perpendicular to the surface of the material, there is a driving mechanism motivated by the pressure of the buoyancy component of the boundary layer, the pressure difference of which promotes the flame spread along the cable [10,23]. The flame height is defined as the length from the bottom of the visible flame to the top, while the flame width is the length of the flame along the cable, i.e., *W_f_* in Figure 2.

**Figure 3:**
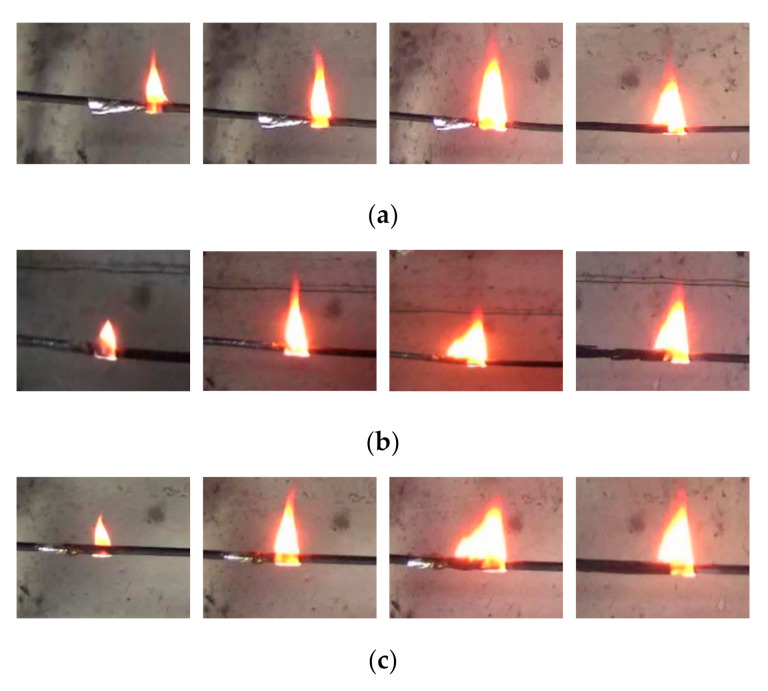
Typical flame shape of a single cable with different core number (flame spreading from left to right). (**a**) The flame shape of two-cores cable; (**b**) the flame shape of three-cores cable; (**c**) the flame shape of four-cores cable.

The flame shapes of a single cable with different core numbers are shown in Figure 3. In the initial stage, the flame wraps the cylindrical cable. The flame transfers heat to the preheating zone through radiation, convection, and conduction. Pyrolysis gas generated from the preheating zone moves under the buoyancy and inertial force, thus the flame height and width gradually increase to form a longer cable combustion area, which results in more and more outer sheaths burning. As combustibles decrease in the combustion area, the flame tail shrinks to form a stable flame, which spreads horizontally along the cable. During the whole flame-spread process, the flame area first decreases and then grows, remaining at a stable state lastly. The flame height first rises and then remains stable, and the flame width first increases and then decreases to a stable value. For polyethylene (PE) wire, He et al. [24] found that during its stable flame-spread stage, the insulation layer melted and the molten insulation material accumulated gradually, and the flame height slowly increased. When the molten material dripped, the flame height was reduced by 56.8%, and the flame width remained basically stable. The difference in changing trends of flame height between the PVC cable and PE wire can be attributed to their different structures. There is a sheath outside the PVC insulation layer, while there is no outer sheath for PE wire. The outer sheath is made of neoprene, which is thermosetting and thus avoids the dripping of the molten PVC. In addition, the change trend of the flame shape for a single cable approximately does not change with the increasing of the core number.

**Figure 4:**
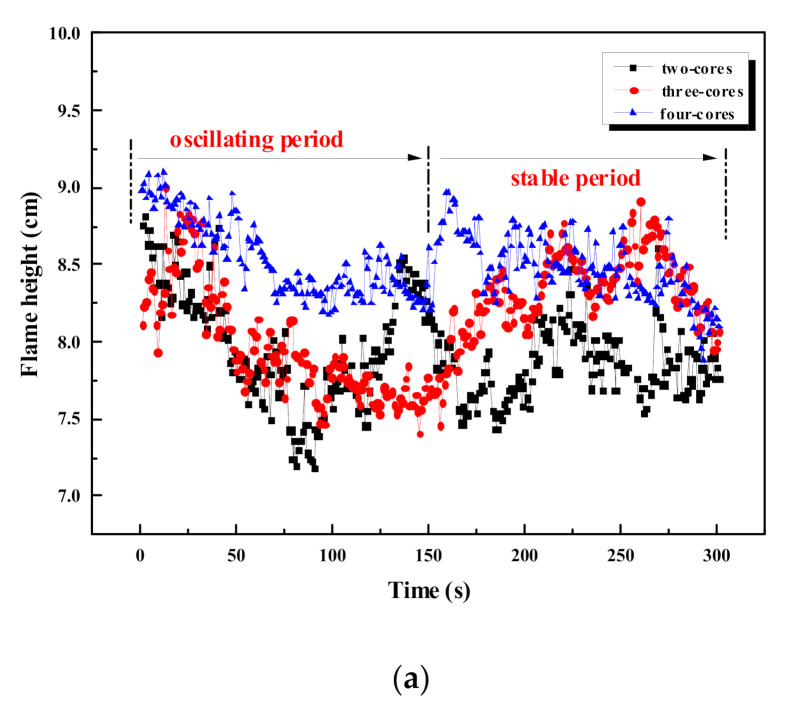

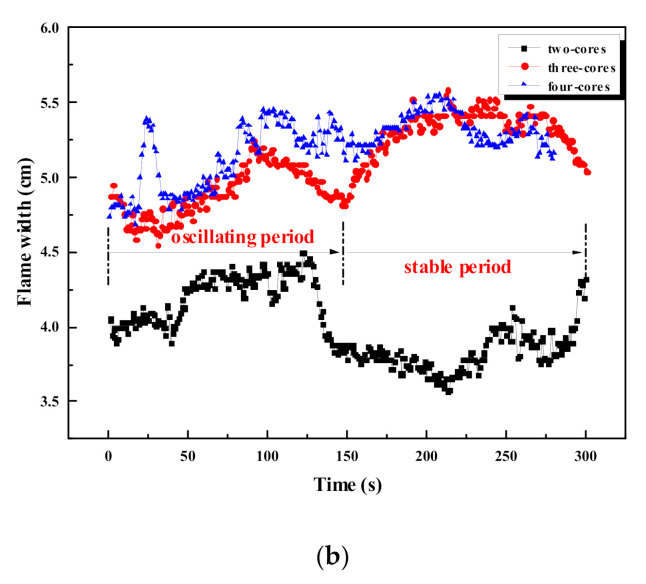
Change of flame size of a single cable with different core numbers. (**a**) Flame height; (**b**) flame width.

The history of flame width and height of a single cable is presented in Figure 4. It is observed in the experiments that the flame is more oscillating during the first 150 s, defined as the first stage of flame spread, while the last 150 s is the second stage. Variance of flame height and width are calculated for the two stages and shown in Table 3. The variance could reflect dispersion degree of the data, and then reflect the fluctuation of flame height and width. It is found that the variance of the first stage is larger than the second stage. Therefore, the first stage is defined as the “oscillating period,” while the second stage is defined as the “stable period.” There are two reasons for the fluctuation of flame height. First, flame height changes dynamically in accordance with the fluctuation of the flame. Second, the outer sheath of the cable peels off during fire and part of the peeling material is burning, which will significantly change the flame height. The average values of the flame width and height during the stable period in Figure 4 are calculated, and the results are presented in Table 4. Table 4 also includes the dimensionless flame height and width, which are defined as the ratio of the flame size to the cable diameter. Compared with the two-core cable, the average flame height and flame width of the three-core cable increase by 4.6 mm and 14.73 mm, respectively. The reason is that the cross-section area of the cable increases as the core number increases, and the mass of the combustible material increases accordingly. Compared with three-core cable, the diameter of the four-cores cable only increases by 1 mm. Therefore, the increase of the flame size is not obvious, i.e., the average flame width increases by 0.1 mm, and the average flame height increases by 1.7 mm.

It can be seen from Table 4 that the dimensionless flame height of a single cable decreases as the diameter of the cable increases. The reason is as follows: the height of the diffusion flame is determined by the buoyancy and the inertial force. The Froude number [25] is usually used to characterize the ratio of inertial force to buoyancy, and its expression is shown in Formula (1).

(1) $$F_{r}{=u}_{0}^{2}/(\mathrm{Wg})$$

(2) $$H_{f}/{W\sim F}_{r}^{n}$$

(3) $$H_{f}/{W\sim W}^{-n}$$

In the above formulas, *W* is the width of the sample, *H_f_* is the height of the flame, and the value range of *n* is 1/5~1/3. When the *F_r_* is small, the flame height is dominated by the buoyancy, and the dimensionless flame height conforms to Formula (3) [26]. Combining the Formulas (2) and (3), Formula (4) can be obtained, which indicates the dimensionless flame height decreases with the increase of sample width. Therefore, when the core number of single cable increases, the cable diameter and the width of the cable increase, and thus the dimensionless flame height of the cable decreases.

**Figure 5:**
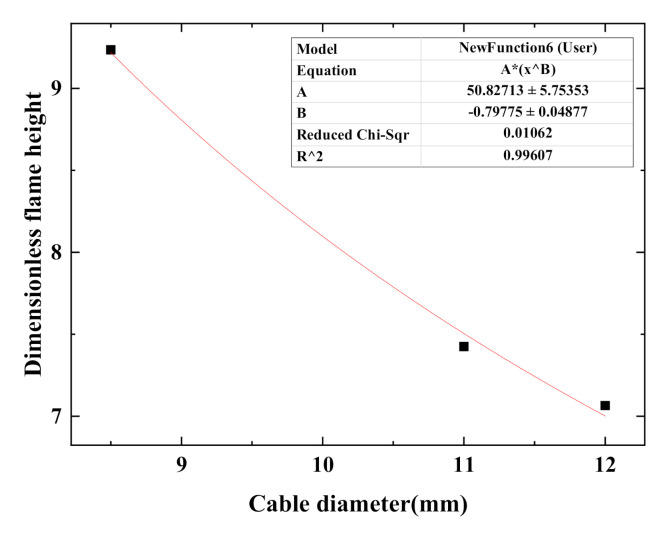
The relationship between the dimensionless flame height of a single cable and the cable diameter.

According to Formula (3), curve fitting concerning the dimensionless flame height of a single cable and the cable diameter is conducted and results are shown in Figure 5. The fitting formula is as follows:

(4) $$H/d=50.83d^{-0.798}$$

**Figure 6:**
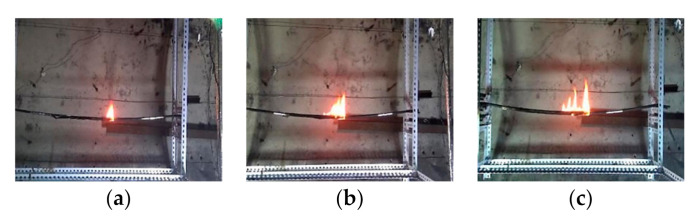
Flame shape of multiple cables with different cable number. (**a**) Single cable; (**b**) two cables; (**c**) three cables.

The paper also studies the influence of cable number on the flammability and flame-spread characteristics of multiple cables when the cable spacing is zero and the number of cable cores is three. The flame shape of multiple cables during the stable period of flame spread is shown in Figure 6. Compared with the flame shape of a single cable, the flame width, flame height, and flame area of multiple cables increase as the cable number increases. As for flame structure, multiple flame bifurcations could be observed, which means the entire flame is divided into some flame branches. The more the number of cables, the more obvious the bifurcation phenomenon could be observed. The uneven surface of multiple cables may cause this phenomenon. In addition, multiple cable burning zones is formed. The spread rate of the pyrolysis front and the cable burning rate are different in different burning zones, leading to the fact that flame front is not straight.

**Figure 7:**
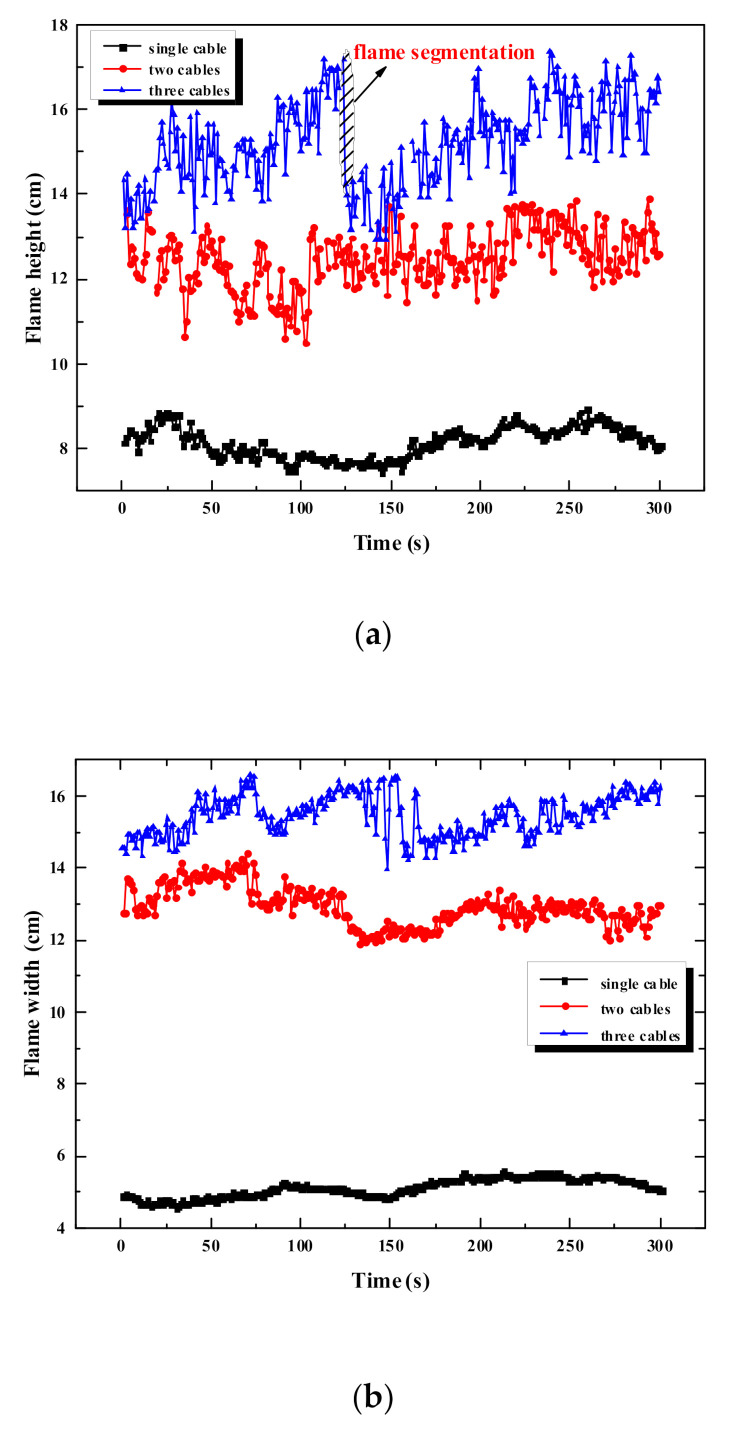
Flame size of multiple cables versus time. (**a**) Flame height; (**b**) flame width.

The history of flame size in the stable stage of flame spread of multiple cables is shown in Figure 7. The change curve of flame size of multiple cables has a larger fluctuation amplitude than the single cable. When the number of cables is three, the fluctuation of the flame size is the most significant. As the number of cables increases, the flame height and flame width gradually increase, while the increment tends to reduce. The reason is that the increase in the number of cables causes more flame branches, resulting in a decrease in the increment of the flame size.

The average value of the flame size is calculated from Figure 7. Further, the variance of flame size under different experimental conditions is calculated. The results concerning the average flame size and its variance are presented in Table 5. As for the flame height, the increment between two cables and a single cable is 41.5 mm, and it is 27.66 mm between three cables and two cables. For the flame width, the increment between two cables and a single cable is 76.3 mm, and it is 25.2 mm between three cables and two cables. It is concluded that with the increase in the number of cables, the flame size increases, but the increment is decreases. Besides, for the flame size of multiple cables, the average flame height is approximately equal to the flame width. The difference between the average flame height and width of the two cables is 4.7 mm, while the difference of the three cables is 2.2 mm. In contrast, the difference between the average flame width and height of a single cable is 29.9 mm. In conclusion, as the number of cables increases, the difference between the flame height and width decreases. The reason is that when multiple cables are arranged in parallel, the width of the total cables increases, and thus the flame height increases. The increase in flame height is smaller than the increase in flame width. Therefore, their difference decreases.

**Figure 8:**
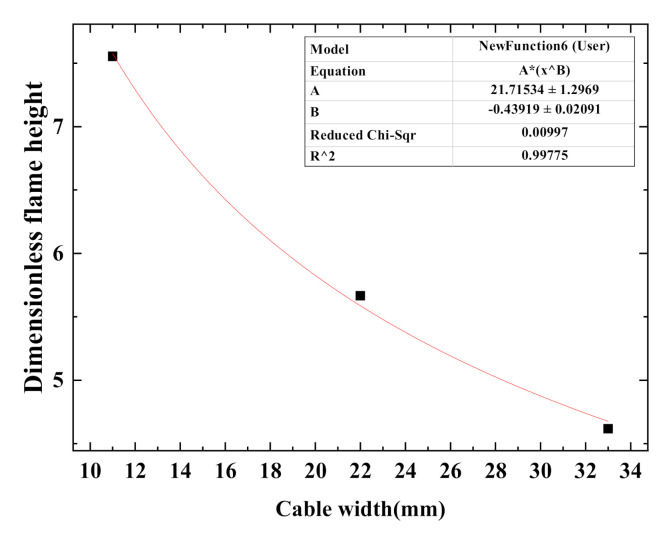
The relationship between the dimensionless flame height and the cable width.

For multiple cables, since the cables are closely arranged and the deformation is small during the combustion process, the multiple cables could be deemed as a plane with a width of *W*. The dimensionless flame height is defined as the ratio of the flame height (*H*) to the total cable width (*W*). Based on Formula (3), curve fitting concerning the dimensionless flame height and the total width of multiple cables is conducted, and results are shown in Figure 8. The fitting formula is:

(5) $$H/W\;=\;21.72{\;W}^{-0.439}$$

As the number of cables increases, the width of the cable rises, and thus the dimensionless flame height of the cable decreases according to Formula (5). This is consistent with the predicted trend of the Formula (3).

### 3.2. Flame-Spread Rate

**Figure 9:**
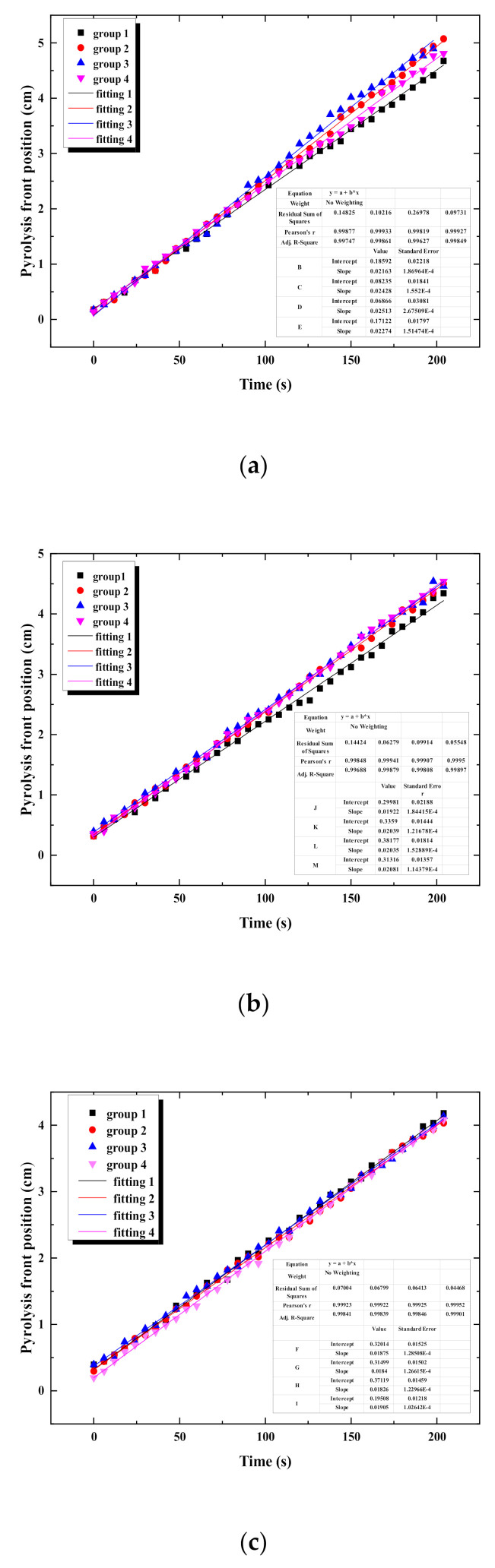
Pyrolysis front position of single cable with different core numbers versus time. (**a**) Two cores; (**b**) three cores; (**c**) four cores.

This paper determines flame-spread rate of PVC cable based on the moving speed of the pyrolysis front. According to the TG-DTG-DSC-MS curve of rubber combustion, the pyrolysis of the rubber starts at about 190 °C, and the pyrolysis rate at this temperature can reach 100% [5,27]. To show the range of the pyrolysis area more clearly, the temperature from 190 °C to 225 °C is selected in the infrared video as the pyrolysis temperature, from which the change of the pyrolysis front position over time can be obtained, as shown in Figure 9. To reduce the experimental error, four curves concerning pyrolysis front position versus time are chosen in the stable flame-spread stage, and the average value of slopes of the fitting lines is calculated. This average value is deemed as the flame-spread rate of the cable. Consequently, the flame-spread rates of a single cable with two-cores, three-cores and four-cores are calculated and the values are 1.406 cm/min, 1.222 cm/min, 1.116 cm/min, respectively.

**Figure 10:**
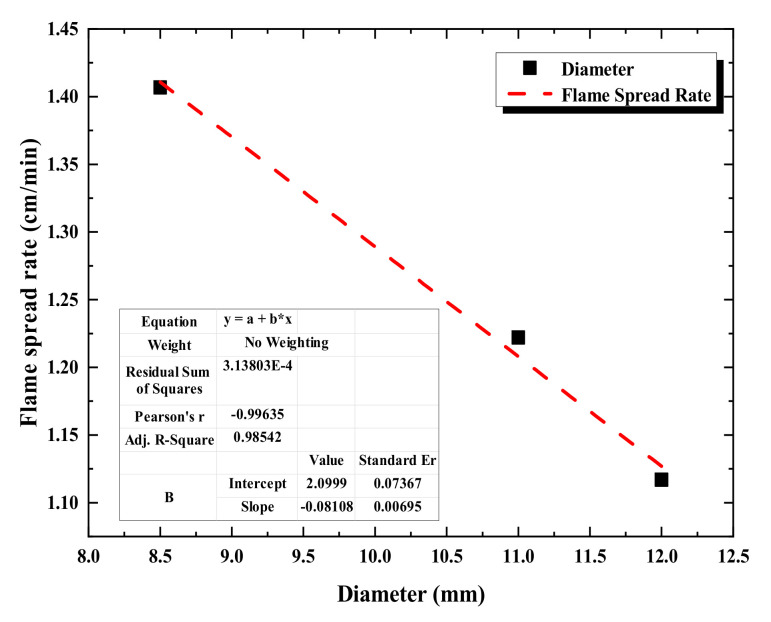
Fitting relationship between the flame-spread rate and diameter of a single cable.

The diameters of a single two-cores three-cores and four-cores cable are 8.5 mm, 11 mm, and 12 mm, respectively. The fitting relationship between the flame-spread rate of the single cable and its diameter is shown in Figure 10, which demonstrates that the cable flame-spread rate decreases linearly with the increase of the diameter.

For the flame spread over solid materials, the entrainment effect on both sides of the material is strong when the sample width is small, and thus the convection heat transfer is dominant. The convective heat flux received by the solid surface can be expressed as [25]:

(6) $${\dot{q}}_{i}=h_{i}(T_{g}-T_{s})$$

(7) $$h_{i}{\sim W}^{-n}$$

In above formulas, *h_i_* is the convective heat transfer coefficient. *T_g_* and *Ts* are the gas temperature near the solid surface and the solid surface temperature, respectively, and *W* is the sample width. The convective heat transfer coefficient shows a power-law attenuation trend as the sample width increases. Therefore, the convective heat transfer decreases with the increase of sample width [28,29]. When the number of cores of a single cable increases, both the thickness and width increase, and thus the convective heat transfer decreases, which causes the flame-spread rate to decrease. Additionally, Higuera [30] obtained the correlation between the horizontal flame-spread rate (*V_f_*) and the radius (*r*) of cylindrical combustibles:

(8) $$V_{f}\propto G_{r}^{\frac{1}{4}}N\frac{k_{s}}{\rho_{s}c_{s}r}$$

where *G_r_* is the Grashof number, and *N* is the ratio of the thickness of the gas boundary to the thickness of the solid boundary. *ks*, *ρ_s_*, *c_s_*, and *r* are the thermal conductivity, density, specific heat capacity, and radius of the cylindrical material, respectively. From Formula (8), it can be deduced that the flame-spread rate decreases as the radius of the cylindrical combustible increases.

**Figure 11:**
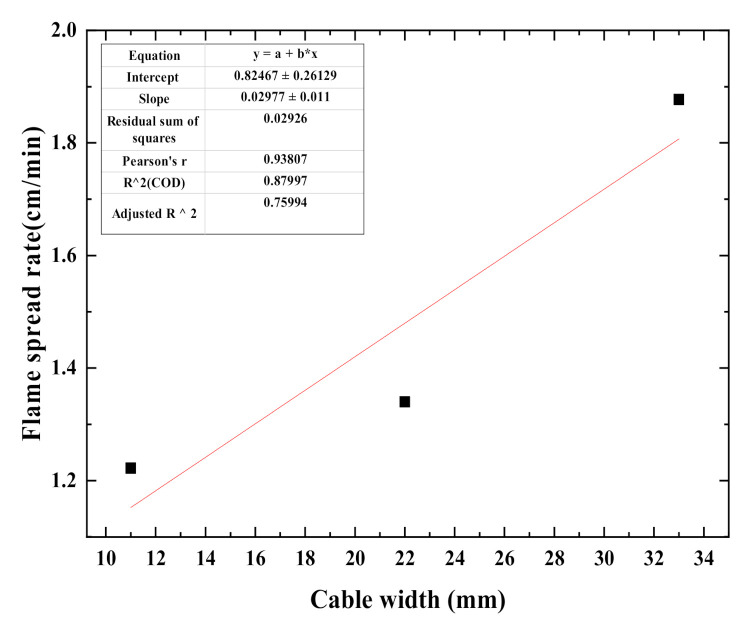
Fitting relationship between cable flame-spread rate and cable width.

For multiple cables, flame-spread rates of single, two, and three cables were calculated and the results were 1.222 cm/min, 1.340 cm/min, 1.877 cm/min, respectively. Since multiple cables are arranged seamlessly, they could be regarded as rubber plates. The width of the plate is positively correlated with the number of cables. The change of flame-spread rate with multiple cables width is presented in Figure 11, which demonstrates that as the width increases, the flame-spread rate also increases.

Zhang [25] proposed that flame-spread rate of solid material is determined by the heat transfer mechanism. In fact, the heat transfer is closely related with heat release rate. Magalie et al. [31] conducted cone calorimeter experiment to investigate the correlation between the number of closely arranged cables and the maximum heat release rate, and proposed a prediction formula:

(9) $${\mathrm{pHRR}}_{1}=2.19N^{1.14}$$

In addition, Magalie et al. compared the predicted results calculated from Formula (9) and the experimental results, verifying the accuracy of the prediction. To achieve the correlation between the number of cables and the maximum heat release rate of each cable, Formula (10) is proposed in this work.

(10) $${\mathrm{pHRR}}_{1}/N=2.19N^{0.14}$$

It is indicted from Formula (10) that the maximum heat release rate of each cable increases with the increase in the number of multiple cables. There are mainly two ways for the heat released to transfer to the preheating zone of cables, namely, gas-phase heat transfer and solid-phase heat transfer (i.e., heat conduction). The former includes convective heat transfer and radiant heat transfer. When the width of the sample is large, compared with the convective heat transfer, the flame radiant heat transfers is dominant, which conforms to the Stenfen–Boltzman law:

(11) $${\dot{q}}_{i}(W)=\varepsilon_{f}{\sigma T}_{f}^{4}F$$

where *ε_f_* is the flame emissivity, *σ* is the Stenfen-Boltzman constant, *T_f_* is the flame height, and *F* is the view factor of the flame to the unburnt surface. According to the experiment, the flame height and width of the cable increase as the number of cables (or cable width) increases, enlarging the view factor. In addition, it can be deduced from the Formula (12) that the flame emissivity also increases with the increase of the cable width.

(12) $$\varepsilon_{f}\approx 1-e^{-\mathrm{KW}}$$

where *K* is the emission coefficient, and *W* is the width of the sample.

The solid-phase heat transfer, i.e., heat conduction to the preheating zone of cables can be expressed as [23]:

(13) $${\dot{q}}_{c}=\frac{T_{c}-T_{p}}{\delta_{s}/\lambda_{s}-r_{c}/\lambda_{c}}$$

where $\delta_{s}$ and $\lambda_{s}$ are the thickness and thermal conductivity of insulating layer, respectively. $r_{c}$ and $\lambda_{c}$ are the radius and thermal conductivity of cable core, respectively. $T_{c}$ is the temperature of cables core, and $T_{p}$ is the pyrolysis temperature. As the number of cables increases, the flame temperature rises [9,32,33]. The temperature of the cable core is positively correlated with the flame temperature. Therefore, the solid-phase heat transfer increases according to Formula (13).

In conclusion, the maximum heat release rate of each cable, solid-phase and gas-phase heat transfer rise with an increase in the cable number, and thus the flame-spread rate increases as the cable number (or total cable width) rises.

### 3.3. Temperature Distribution

**Figure 12:**
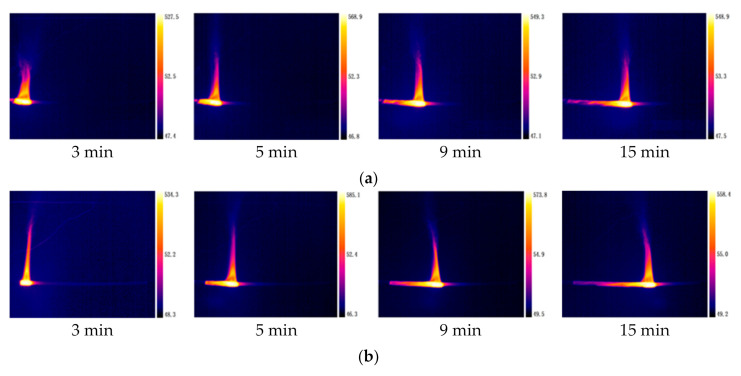

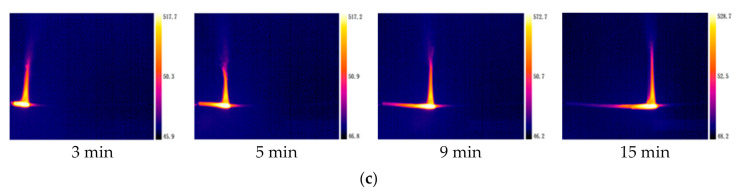
Temperature distribution of a single cable with different core number at different time. (**a**) two-cores; (**b**) three-cores; (**c**) four-cores.

**Figure 13:**
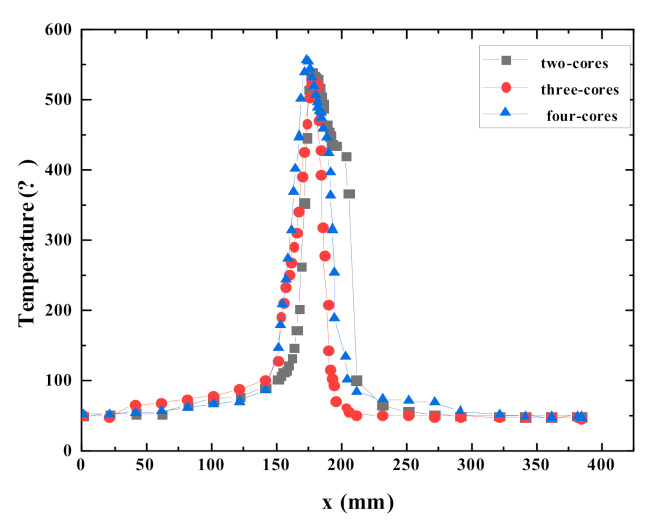
Surface temperature distribution in the core area of a single cable during flame spread.

Temperature distribution plays an important role in flammability of combustibles and flame spread [10,14,34]. In this work, an infrared camera placed in front of the cables rather than at the top of the cables are employed to capture the temperature distribution of cable surface and flame. Thus, the temperature distribution in the front of the cables is obtained, while the temperature distribution on the top of the cables is not obtained. The surface temperature distribution of a single PVC cable at different time is shown in Figure 12. It can be seen that the highest temperature of the cable surface is found in the core area. The flame temperature increases first and then decreases from the bottom to the top. When the combustion area is in the middle of the cable, the temperature distribution at various positions on PVC cable surface is shown in Figure 13. In the burnout area, the closer to the flame zone, the higher the temperature. In the unburnt area, as the distance from the flame zone increases, the cable temperature decreases sharply, which is different from the results of wire fire experiment conducted by Zhang et al. [35]. In Zhang et al.’s experiment, as the distance between the unburnt surface of the wire and the flame zone increases, the temperature decrease is relatively slow. The reason is that Zhang et al. electrified the wire in the experiment, and the heating effect of the current caused the temperature of the unburnt area higher than that of the cable in this paper. Since the temperature distribution of the burning cable is in dynamic change, the highest temperature of the cable surface is different at different time. The average values of the highest temperature of the two-cores, three-cores, and four-cores cables are calculated and the results are 554.8 °C, 548 °C, 555.2 °C, respectively. This is different from the results of Huang et al. [2,36], who found that the average value of highest temperature rises as the number of cores increases. The difference in results could be attributed to the difference in experimental conditions. Huang et al. conducted the vertically cable flame-spread tests in confined compartments and horizontal cable tray fire tests in the open space. However, this work focuses on flame spread over horizontal cables in a confined compartment.

**Figure 14:**
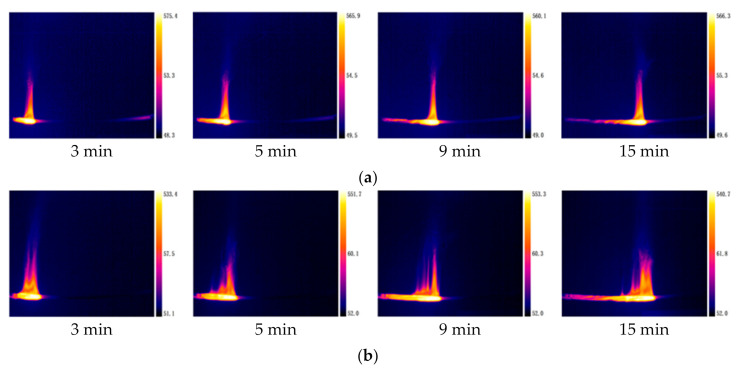

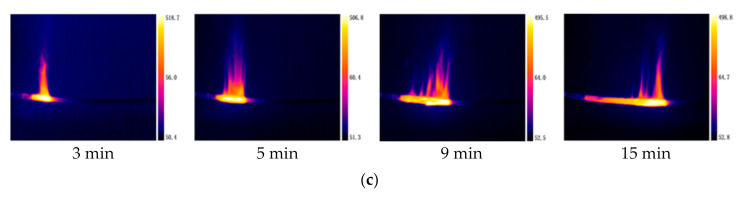
Temperature distribution of multiple cables with different cable number at different time. (**a**) Single cable; (**b**) two cables; (**c**) three cables.

The temperature distribution of multiple PVC cables at different time is shown in Figure 14. As the number of cables increases, the cable temperature distribution range expands, and the length of the pyrolysis and combustion area increases. There are multiple bright spots in the cables’ core area, indicating that the maximum temperature distribution of the core is uneven. The reason is that compared to a single cable flame, the flame of multiple cables has multiple burning areas, which leads to multiple high temperatures areas. The temperature distribution of multiple cables is also in dynamic change. The average values of the highest surface temperatures of single, two, and three cable in fire are 548 °C, 533 °C, and 479 °C, respectively.

## 4. Conclusions

Experimental study on flammability and flame-spread characteristics of polyvinyl chloride (PVC) cable is conducted in this work. Flame shape, flame size, flame-spread rate, and temperature distribution are obtained, and the effects of core number of single cable and cable number of multiple cables on the experimental results are discussed. Correlation analysis concerning cable structure, cable number, flammability, and flame-spread characteristics of the PVC cable is conducted. The main conclusions are presented as follows.

(1) The changing trend of flame shape of single cable does not vary with cable core number. The fluctuation range of flame size of multiple cables is larger than that of the single cable. For both single cable and multiple cables, as the cable core number (or cable number) increases, average flame height and width increase while the increment decreases, and the difference between the average flame height and width decreases. Fitting formulas concerning dimensionless flame height and single cable diameter (or total width of multiple cables) are obtained. The former is negatively correlated with the latter.

(2) For single cable, the flame-spread behavior is determined by convective heat transfer, which decreases as the cable core number increases. Therefore, the flame-spread rate decreases as the cable core number increases. For multiple cables, flame-spread behavior is dominated by radiative and conductive heat transfer, which increases as the cable number increases. Therefore, the flame-spread rate increases as the cable number increases.

(3) For single cable, the flame temperature rises first and then drops as the vertical distance from the flame bottom increases. The maximum temperature of cable surface, which decreases first and then increases as cable core number increases, is found in the cable core area. For multiple cables, with an increase in cable number, the length of pyrolysis and combustion zone increases while the maximum temperature of cable surface decreases.

Results obtained in this work are beneficial to fire hazard evaluation and safety design of PVC cables used in cities or factories. Moreover, this work provides an in-depth understanding of the relationship among the cable structure, cable number, and flammability of cable materials.

## Figures and Tables

**Table 1:** Physical parameters of cable materials.

Materials	Thermal Conductivity W/(m·K)	Density g/cm^3^	Specific Heat Capacity kJ/(kg·K)
Copper	387	8940	0.38
Neoprene	0.19	1250	1.7
PVC	0.2	1500	1.5

**Table 2:** Experimental conditions.

Condition Number	Core Number	Number of Cables
1	two-cores	1
2	three-cores	1
3	four-cores	1
4	three-cores	1
5	three-cores	2
6	three-cores	3

**Table 3:** Variance of flame height and width for cables with different cores in different periods.

Variance	Two-Cores		Three-Cores		Four-Cores	
	Oscillating Period	Stable Period	Oscillating Period	Stable Period	Oscillating Period	Stable Period
Flame height variance	0.153449	0.044898	0.131187	0.07525	0.062321	0.043459
Flame width variance	0.033124	0.020265	0.026224	0.021472	0.051584	0.011915

**Table 4:** Average flame size of a single cable with different core numbers.

Core Numbers	Two-Cores	Three-Cores	Four-Cores
Average flame height (mm)	78.494	83.096	84.779
Average flame width (mm)	38.387	53.113	53.216
Dimensionless flame height	9.235	7.554	7.065
Dimensionless flame width	4.516	4.828	4.435

**Table 5:** Average flame size of multiple cables with different cable number.

Numbers of Cables	Single	Two	Three
Average flame height (mm)	83.09	124.64	152.35
Average flame width (mm)	53.11	129.38	154.53
Variance of flame height	0.130447	0.489874	0.173649
Variance of flame width	0.066659	0.294971	0.318655
